# A Simple and Low-Cost Strategy to Improve Conidial Yield and Stress Resistance of *Trichoderma guizhouense* through Optimizing Illumination Conditions

**DOI:** 10.3390/jof8010050

**Published:** 2022-01-05

**Authors:** Yifan Li, Xiya Meng, Degang Guo, Jia Gao, Qiwei Huang, Jian Zhang, Reinhard Fischer, Qirong Shen, Zhenzhong Yu

**Affiliations:** 1 Laboratory of Bio-Interactions and Crop Health, Jiangsu Provincial Key Lab for Organic Solid Waste Utilization, National Engineering Research Center for Organic-Based Fertilizers, Jiangsu Collaborative Innovation Center for Solid Organic Waste Resource Utilization, Nanjing Agricultural University, Nanjing 210095, China; liyifan@njau.edu.cn (Y.L.); 2020803234@stu.njau.edu.cn (X.M.); 2020103137@njau.edu.cn (D.G.); qwhuang@njau.edu.cn (Q.H.); zhangjian@njau.edu.cn (J.Z.); qirongshen@njau.edu.cn (Q.S.); 2 Department of Microbiology, Institute for Applied Biosciences, Karlsruhe Institute of Technology (KIT)—South Campus, 76131 Karlsruhe, Germany; jia.gao@kit.edu (J.G.); reinhard.fischer@kit.edu (R.F.)

**Keywords:** biofertilizer, light intensity, light wavelength, conidial yield, conidial stress resistance

## Abstract

Light is perceived by photoreceptors in fungi and further integrated into the stress-activated MAPK HOG pathway, and thereby potentially activates the expression of genes for stress responses. This indicates that the precise control of light conditions can likely improve the conidial yield and stress resistance to guarantee the low cost and long shelf life of *Trichoderma*-based biocontrol agents and biofertilizers. In this study, effects of wavelengths and intensities of light on conidial yield and stress tolerance to osmotic, oxidative and pH stresses in *Trichoderma guizhouense* were investigated. We found that 2 μmol photons/(m^2^ × s) of blue light increased the conidial yield more than 1000 folds as compared to dark condition and simultaneously enhanced conidial stress resistance. The enhanced conidial stress resistance is probably due to the upregulated stress-related genes in blue light, which is under the control of the blue light receptor BLR1 and the MAP kinase HOG1.

## 1. Introduction

Fungi adapt continuously to the ever-changing environment to survive in nature. Light, as one of the most common environmental cues, regulates various morphological and physiological processes of fungi [1]. In *Aspergillus nidulans*, red light stimulates conidiation (asexual development) and represses the formation of cleistothecia (sexual development) [2,3,4], whereas in *Neurospora crassa* and *Trichoderma atroviride* blue light promotes conidiation [5,6]. However, in the plant pathogen *Alternaria alternata*, blue and green light repress conidiation [7]. Sporangiophores of the zygomycete fungus *Phycomyces blakesleeanus* exhibit phototropism in response to blue light [8]. White light also accelerates biofilm formation and melanin accumulation in *A. niger* [9]. Hence, light responses vary from fungus to fungus.

Fungi utilize different photoreceptors to perceive a wide range of light wavelengths [1,6,10,11]. The most representative fungal photoreceptors studied at the molecular level are the light, oxygen, and voltage (LOV) domain-containing blue-light receptor white collar 1 (WC-1) and vivid (VVD) in *N. crassa*, and the red-light receptor phytochrome FphA in *A. nidulans*. The blue- and red-light signaling pathways have been well studied in *N. crassa* and *A. nidulans*, respectively [1,5,12]. In addition, fungi can also sense blue light with cryptochrome and green light with the retinal-binding protein opsin [13,14,15,16,17]. On the other hand, although responses of many fungi to monochromatic light, which is perceived by specific photoreceptors, have been studied, the effects of the light intensities of specific wavelength on fungal development have received comparatively little attention.

*Trichoderma* species are widely used in agriculture as biocontrol agents and biofertilizers because of their outstanding performances in antagonizing plant pathogenic fungi and promoting plant growth [18,19]. *T. guizhouense*, initially isolated from mature compost, defeats plant pathogenic fungi through reactive oxygen species (ROS) and secondary metabolites [20,21]. It can also secrete the swollenin protein to benefit plant growth [22]. These properties make *T. guizhouense*-based biocontrol agents and biofertilizers increasingly popular. In the production of these agents and biofertilizers, high conidial yield and stress resistance are the guarantees of cost reduction and shelf-life extension.

In the entomopathogenic fungus *Metarhizium robertsii*, light modulates the expression of stress-related genes differentially and therefore affects conidial tolerance [23]. We also proved previously that in *A. alternata* white light regulates the expression of catalase-encoding genes [7]. Recently, we provided evidence that the blue light receptor BLR1, the white collar 1 orthologue, dominates conidiation in *T. guizhouense* and the blue light receptor ENV1, the orthologue of VVD, modulates photoadaptation [24]. BLR1 employs the stress-activated MAPK HOG pathway, which is also responsible for the sensing of osmotic, oxidative, and heat-shock stresses in *A. nidulans*, to activate a large proportion of blue light-regulated genes [24,25,26,27,28,29]. However, how other monochromatic light affects conidiation in *T. guizhouense* remains unknown.

Here, we investigated the effects of light wavelengths and intensities on the conidial yield and resistance to several abiotic stresses in *T. guizhouense*. The mechanism for the enhancement of stress resistance of conidia produced in light was also elucidated.

## 2. Materials and Methods

### 2.1. Strains and Culture Conditions

Wild type of *Trichoderma guizhouense* NJAU 4742 and three mutants, *Δblr1*, *Δenv1* and *Δhog1*, which were constructed previously [24], were used in this study. The blue light receptors BLR1 and ENV1 are absent in the *Δblr1*- and the *Δenv1*-mutant strains, respectively. The *Δhog1*-mutant strain lacks the gene encoding MAP kinase HOG1. All strains were cultivated on potato dextrose agar medium (PDA, BD Difco, Germany) at 28 °C. pH, osmotic and oxidative stresses imposed or illumination conditions during the cultivation were indicated additionally in the following experiments.

### 2.2. Light System

The light system used in this study was developed previously [30]. The LED panel hanged in the metal box is 39 cm long and 28 cm wide and emits blue (450 nm), green (525 nm), red (700 nm), far-red (760 nm) and white light. LEDs soldered on the panel were purchased from Ushio Inc. (Tokyo, Japan). The quality of the LEDs was measured by JAZ-COMBO S/N:JAZA0503 with a QP400-1-VIS-NIR and CC-3-UV-S spectrometer unit (Ocean Optics). The wavelengths, intensities and specific timings of light were automatically controlled by the self-made software coded for Photon P1 WIFI module. An ocean optics JAZA0503 spectrometer was used to calibrate the software. The photon flux of each LED type was measured at a distance of 20 cm and the maximum photon flux was limited to 17 µmol photons/(m^2^ × s). Six light boxes were placed in an incubator in the dark room to make sure that the conditions except for illumination in each box are identical.

### 2.3. Quantification of Conidia

Conidial yield was quantified as previously described by Li et al. [24]. The wild type strain was revived on PDA medium for two days at 28 °C in the dark and afterwards fresh mycelia were inoculated on PDA medium (Ø 6 cm petri dish) using a hole puncher. The inoculated plates were incubated in darkness or different light conditions at 28 °C for four days in light boxes. Conidia of each plate were harvested with 20 mL distilled water and the suspension was filtered with Miracloth (Millipore, Merck KGaA, Darmstadt, Germany) to remove the mycelia. The filtrate was diluted properly, and then conidial concentration was determined using a hemocytometer. Three biological replicates of each culture condition were analyzed, and the assay was repeated three times.

### 2.4. Assessment of Conidial Stress Resistance

Assessment of conidial resistance to abiotic stresses was performed as previously described by Dias et al. with minor modifications [23,31]. Conidia produced in different wavelengths or light intensities were harvested as above and spore suspensions were diluted to a final concentration of 1 × 10^3^ conidia/mL. 100 μL of a spore suspension was spread on PDA medium (Ø 9 cm petri dish) supplemented with different concentrations of NaCl (0, 0.2, 0.4, 0.6, 0.8 and 1.0 M), KCl (0, 0.2, 0.4, 0.6, 0.8 and 1.0 M) and sorbitol (0, 0.4, 0.8, 1.2 and 1.6 M). Oxidative stress was imposed with different concentrations of hydrogen peroxide (0, 1, 2 and 3 mM). To analyze the conidial resistance to different pH, the pH values of the PDA medium were adjusted to 6.0, 7.0, 8.0, 9.0 and 10.0 before autoclaving. The germination rate was then calculated after 30 h of incubation at 28 °C in the dark. Each culture condition has three biological replicates and the experiment was performed in triplicate.

### 2.5. RNA Isolation and Quantitative Real-Time PCR (qPCR)

The wild type, the *Δblr1*-, the *Δenv1*-, and the *Δhog1*-mutant strains were incubated on the PDA medium (Ø 6 cm petri dish) covered with cellophane at 28 °C in the dark for 24 h. The mycelium was harvested in dim-green light immediately or after 45 min illumination of blue light and frozen immediately in liquid nitrogen. Mycelia were disrupted in a homogenizer (Precellys^®^ Evolution Super Homogenizer, Bertin, France) with glass beads at 5500 rpm for 2 min. Total RNA was extracted using the SteadyPure^®^ Plant RNA Extraction Kit (Accurate Biotechnology (Hunan) Co., Ltd., Shenzhen, China). ~1 μg of total RNA was used for cDNA synthesis with HiScript^®^ II Q RT SuperMix for qPCR (+gDNA wiper) (Vazyme Biotechnology Co., Ltd., Nanjing, China). The cDNA synthesis included 2 min of the removal of gDNA at 42 °C, followed by 15 min at 50 °C for the reverse transcription reaction. The cDNA samples were diluted to a final concentration of 100 ng/μL in DEPC water for qPCR.

qPCR was performed with the ChamQ SYBR qPCR Master Mix (Vazyme Biotechnology Co., Ltd., Nanjing, China). Each reaction of 20 μL contained 10 μL of 2 × ChamQ SYBR qPCR Master Mix, 0.2 μM of primers and 100 ng of cDNA. The program started with 3 min of the inactivation of the reverse transcription reaction at 95 °C, followed by 40 cycles of PCR reaction (10 s at 95 °C and then 30 s at 60 °C). After each PCR, melting curve analyses were carried out to assess the dissociation characteristics of double-stranded DNA. The translation elongation factor 1 alpha (*tef1*) gene (OPB38715) was used to normalize the expression level of each gene. Primers used in this study are listed in Supplementary Table S1. Each expression level is the average of three biological replicates. 

### 2.6. Transcriptome Analysis

Transcriptome data of *T. guizhouense* wild type and the mutants *Δblr1*, *Δenv1* and *Δhog1* in response to blue light are available online in the NCBI database Sequencing Read Archive (SRA) under the accession number PRJNA743899. The genes encoding proteins putatively involved in stress resistance were screened from the differentially expressed genes (DEGs) identified in a previous study [24]. The reliability of the transcriptome data was validated by qPCR. The heatmap was generated in the R environment.

### 2.7. Statistical Analysis

Data are generally presented as means ± S.D. (standard deviation) with the biological replicates as indicated in each figure legend. The statistical analysis was carried out using One-way ANOVA analysis in the IBM SPSS Statistics 25 program.

## 3. Results

### 3.1. Specific Wavelengths of Light Activate Conidiation of T. guizhouense

**Figure 1:**
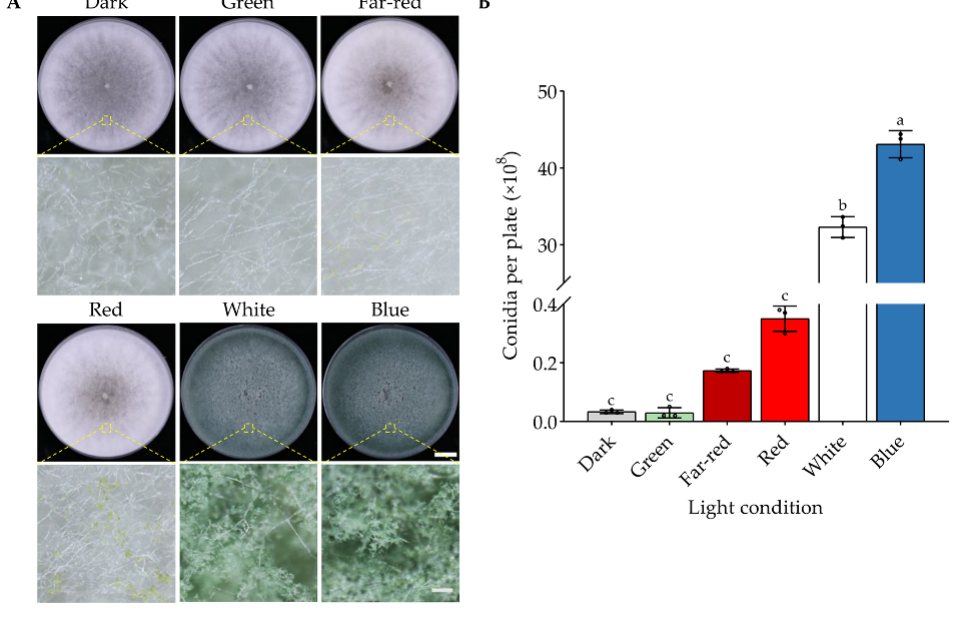
Effects of different wavelengths of light on *T. guizhouense* conidiation. (**A**) Phenotype of the wild type strain cultured under different wavelengths of light. The wild type strain was grown on PDA medium at 28 °C for four days. Scale bar, 1 cm. Colony surface was zoomed in under a stereoscopic micro-scope and the scale bar is 300 μm. (**B**) Quantification of conidia produced under different wavelengths of light after four days of cultivation. The mean values for the three biological replicates are displayed. The error bar represents the standard deviation (SD) of three biological replicates. One-way ANOVA was used for the statistical analysis (*p* ≤ 0.05). Means significantly different from each other do not share the same letter.

Fungi potentially respond to UV, blue, green, red, far-red, and white light, depending on their specific photoreceptors or if the light condition is stressful for them [1]. Hence, we first analyzed the effects of blue, green, red, far-red, and white light on the development of *T. guizhouense* using a self-designed light system. We cultured *T. guizhouense* wild type on PDA medium at 28 °C in blue (450 nm), green (525 nm), red (700 nm), far-red (760 nm) and white light and also in darkness. The light intensity was set to 1.7 μmol photons/(m^2^ × s). After four days of cultivation, the colonies grown in white and blue light were covered with green conidiophores, whereas in green light and darkness the colonies stayed white and only few conidiophores were observed (Figure 1A). Red and far-red light also promoted the formation of conidiophores to some extent.

The conidial yield for each condition was further quantified (Figure 1B). In darkness and green light, the strain produced equal amounts of conidia (0.3 × 10^7^ conidia/plate) suggesting that *T. guizhouense* does not respond to green light as far as the conidiation is concerned. The conidial yield (4.3 × 10^9^ conidia/plate) in blue light was the highest and 1433 folds of that in darkness. Compared with white light (3.2 × 10^9^ conidia/plate), blue light still increased the conidial yield by 34.4%. Conidial yields in red light (3.5 × 10^7^ conidia/plate) and far-red light (1.7 × 10^7^ conidia/plate) were also far lower than that in blue light.

### 3.2. Low Intensity Blue Light Can Promote Conidial Yield Effectively

**Figure 2:**
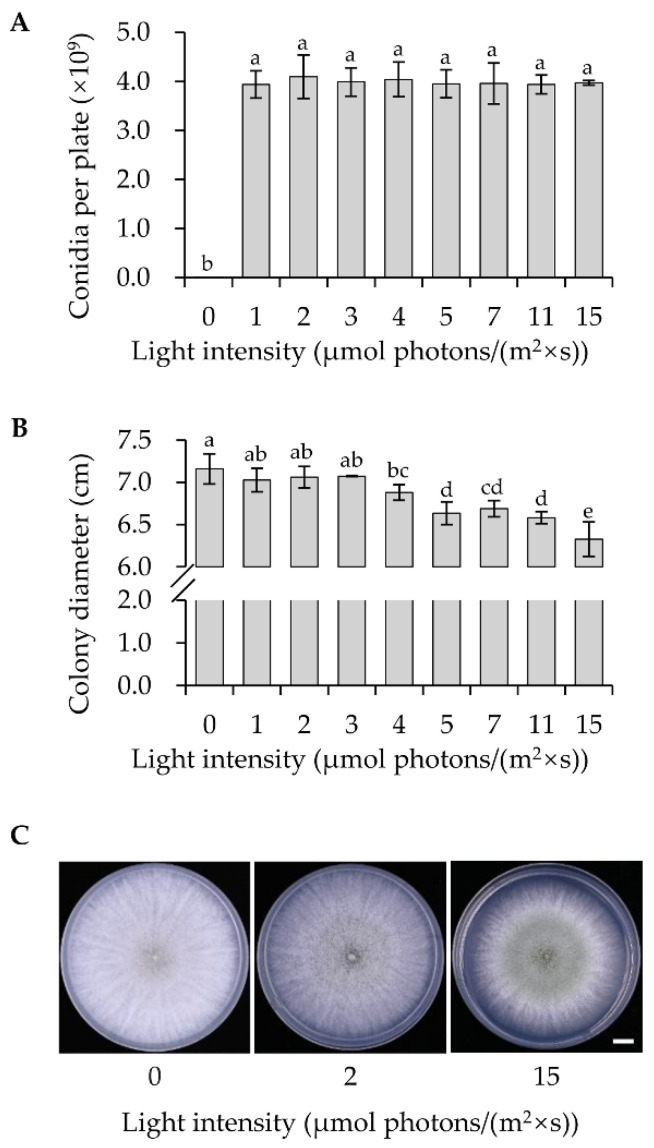
Effects of the intensities of blue light on vegetative growth and conidiation. (**A**) Quantification of the conidia produced in different intensities of blue light. Wild type was grown on the PDA medium at 28 °C for four days. The error bar represents the SD of three biological replicates. (**B**) Colony diameter of wild type in different intensities of blue light. The error bar represents the SD of three biological replicates. One-way ANOVA was used for the statistical analysis (*p* ≤ 0.05). Means significantly different from each other do not share the same letter. (**C**) Phenotype of wild type grown in low and high intensities of blue light. The wild type strain was grown on the PDA medium at 28 °C for two days. Scale bar, 1 cm.

Next, we analyzed the effects of blue-light intensities on *T. guizhouense* conidiation. The intensities of light inside the lightboxes were set to 0, 1, 2, 3, 4, 5, 7, 11 and 15 μmol photons/(m^2^ × s) separately. Wild type was cultured in light boxes for four days and then the conidial yield was quantified. We found that although the conidial yield (4.1 × 10^9^ conidia/plate) in 2 μmol photons/(m^2^ × s) blue light was the highest, the differences between tested intensities were not significant (Figure 2A). Notably, the vegetative growth of the strain was inhibited when the intensity was equal to or more than 4 μmol photons/(m^2^ × s) (Figure 2B). The diameter of the colony under the light intensity of 4 μmol photons/(m^2^ × s) was inhibited by 4% in comparison to that in darkness. The diameters of colonies further decreased along with the increase of the light intensities. It is also notable that strong blue light (15 μmol photons/(m^2^ × s)) activated the conidiation process earlier than the low-intensity blue light (2 μmol photons/(m^2^ × s)) (Figure 2C). After 2 days of cultivation, the colony grown in strong blue light was smaller but much greener than the ones in darkness and in low-intensity blue light.

### 3.3. Blue and White Light Significantly Enhance the Conidial Resistance to Oxidative and pH Stresses

**Figure 3:**
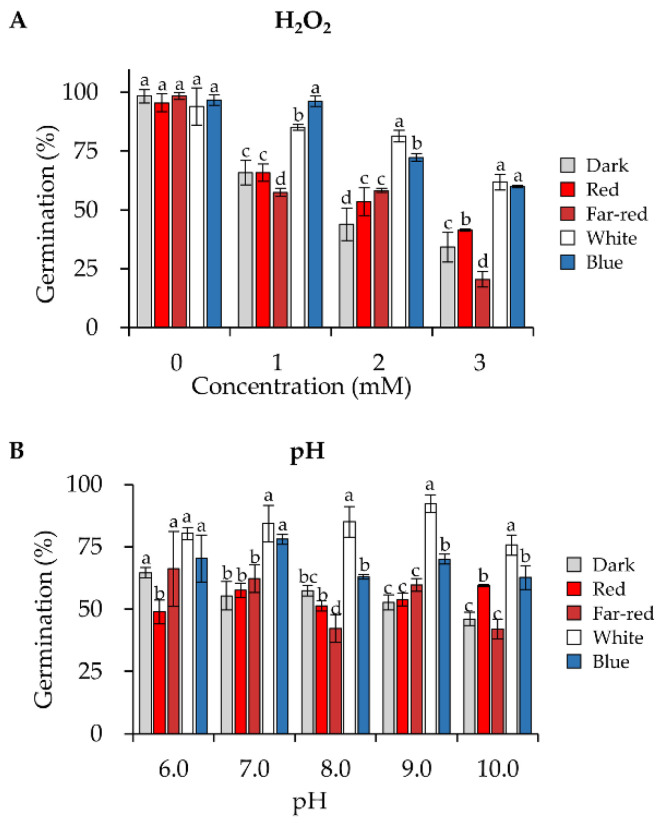
Tolerance to oxidative stress and high pH of conidia formed under different wavelengths of light. Germination rates of conidia under oxidative (**A**) or pH (**B**) stresses. For all experiments, conidia were spread on the PDA medium with increasing concentrations of H_2_O_2_ or pH as indicated, and the germination rates were calculated after 30 h of incubation in the dark. Error bars indicate SD of three biological replicates. One-way ANOVA was used for the statistical analysis (*p* ≤ 0.05). Means significantly different from each other do not share the same letter.

To analyze the conidial stress resistance, conidia produced in darkness or blue, red, far-red, or white light were harvested, and fresh conidia were directly used for the following experiments. To estimate the oxidative stress resistance, conidia were spread on PDA medium with increasing concentrations (up to 3 mM) of hydrogen peroxide. The germination rate of the conidia on each plate was calculated after 30 h of incubation in the dark. At all tested concentrations of hydrogen peroxide, conidia produced under blue or white light were more tolerant to oxidative stress than those produced under other conditions (Figure 3A). After the concentration of H_2_O_2_ was increased to 3 mM, the germination rate of conidia formed under blue light (60.0% germination rate) was about 1.8 folds of that of conidia generated in the dark (34.2%), 1.4 folds of that produced under red light (41.4%), and 2.9 folds of that under far-red light (20.6%).

We also assessed the resistance of conidia to different pH, ranging from 6.0 to 10.0 and conidia formed in white and blue light still exhibited increased tolerance to different pH (Figure 3B). When the pH was above 7.0, conidia produced under white light were more tolerant than conidia produced under blue light.

### 3.4. Blue and White Light Enhance the Conidial Stress Resistance to Several Osmotic Stresses

**Figure 4:**
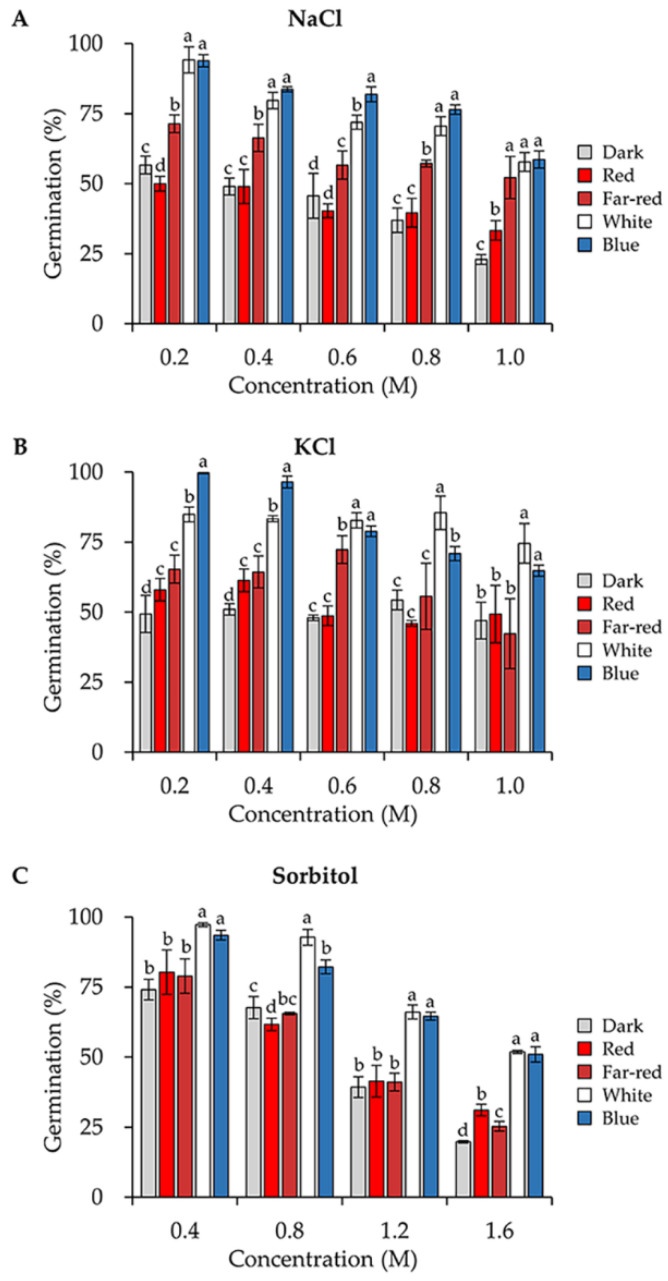
Osmotic stress tolerance of conidia formed under different wavelengths of light. Conidia were spread on the PDA medium with increasing concentrations of NaCl (**A**), KCl (**B**) or sorbitol (**C**) as indicated, and the germination rates were calculated after 30 h of incubation in the dark. Error bars indicate SD of three biological replicates. One-way ANOVA was used for the statistical analysis (*p* ≤ 0.05). Means significantly different from each other do not share the same letter.

To determine the osmotic stress resistance, we first analyzed the germination rates of the conidia produced under different light when incubated on PDA medium supplemented with different concentrations of NaCl. In comparison to darkness, blue and white light significantly enhanced the conidial tolerance towards NaCl (Figure 4A). When the concentration of NaCl increased to 1 M, the germination rate of conidia formed under blue light (58.65%) was 2.5 folds of that in the dark (23.0%). Conidia formed in red light exhibited similar stress resistance to that in the dark, whereas conidia formed in far-red light were more tolerant than that in red light. We also exposed the conidia to different concentrations of KCl and sorbitol. Likewise, conidia formed in blue and white light were always the most tolerant to these stresses (Figure 4B,C).

### 3.5. Blue Light Upregulates the Expression of the Genes Encoding HOG Pathway Components

**Figure 5:**
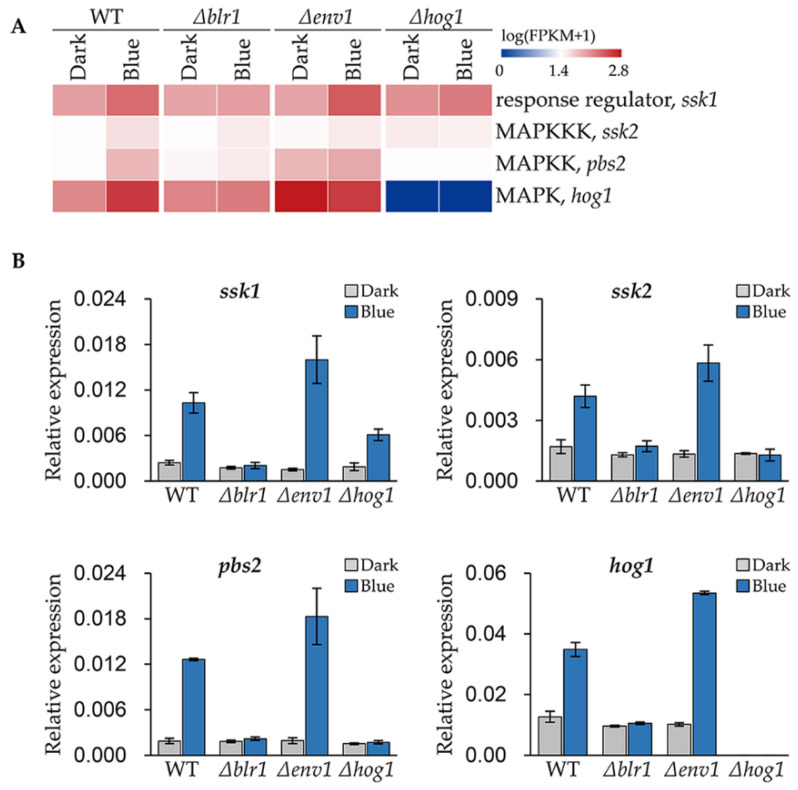
Analysis of transcript abundances of the HOG pathway genes in different strains. (**A**) Transcriptional abundances of the HOG pathway genes derived from the transcriptome data. (**B**) Expression levels of the HOG pathway genes in wild type, the *Δblr1*-, the *Δenv1*- and the *Δhog1*-mutant strains under blue light. The expression level of each gene was normalized to the *tef1* gene. Error bars indicate SD of three biological replicates.

MAPK HOG pathway is crucial for blue light signaling in *T. guizhouense* [24]. By reanalyzing the transcriptome published previously [24], we found that transcript abundance of *ssk2* (OPB39222), *pbs2* (OPB41576) and *hog1* (OPB38173) encoding components of HOG pathway were all upregulated after growth under blue light (Figure 5A). Moreover, the transcript abundance of *ssk1* (OPB38780) encoding an orthologue of *A. nidulans* SskA, the response regulator of the two-component system, was also significantly upregulated. This means in blue light the signaling pathway is probably more sensitive to environmental stresses.

To validate the reliability of the transcriptome data and further analyze the regulatory pattern of the photoreceptors on these genes, we measured the expression levels of these genes in wild type, the *Δblr1*-, the *Δenv1*- and the *Δhog1*-mutant strains by real-time quantitative PCR (qPCR). In wild type, the expression levels of *ssk1*, *ssk2*, *pbs2* and *pbs2* in blue light were 4.3, 2.5, 6.7 and 2.8 folds of that in the dark respectively (Figure 5B). And the fold changes of *ssk1*, *ssk2*, *pbs2* and *pbs2* in the *Δenv1*-mutant strain were 10.6, 4.4, 9.6 and 5.2, respectively, which were higher than that in wild type. However, these genes could not be induced anymore in the *Δblr1*-mutant strain. In the *Δhog1*-mutant strain, the expression levels of *ssk2, pbs2* and *hog1* did not change in blue light. Although *ssk1* was upregulated 3.2 folds after blue light exposure, the expression level was lower than that in wild type.

### 3.6. Blue Light Receptors Regulate the Stress Responsive Genes of T. guizhouense

**Figure 6:**
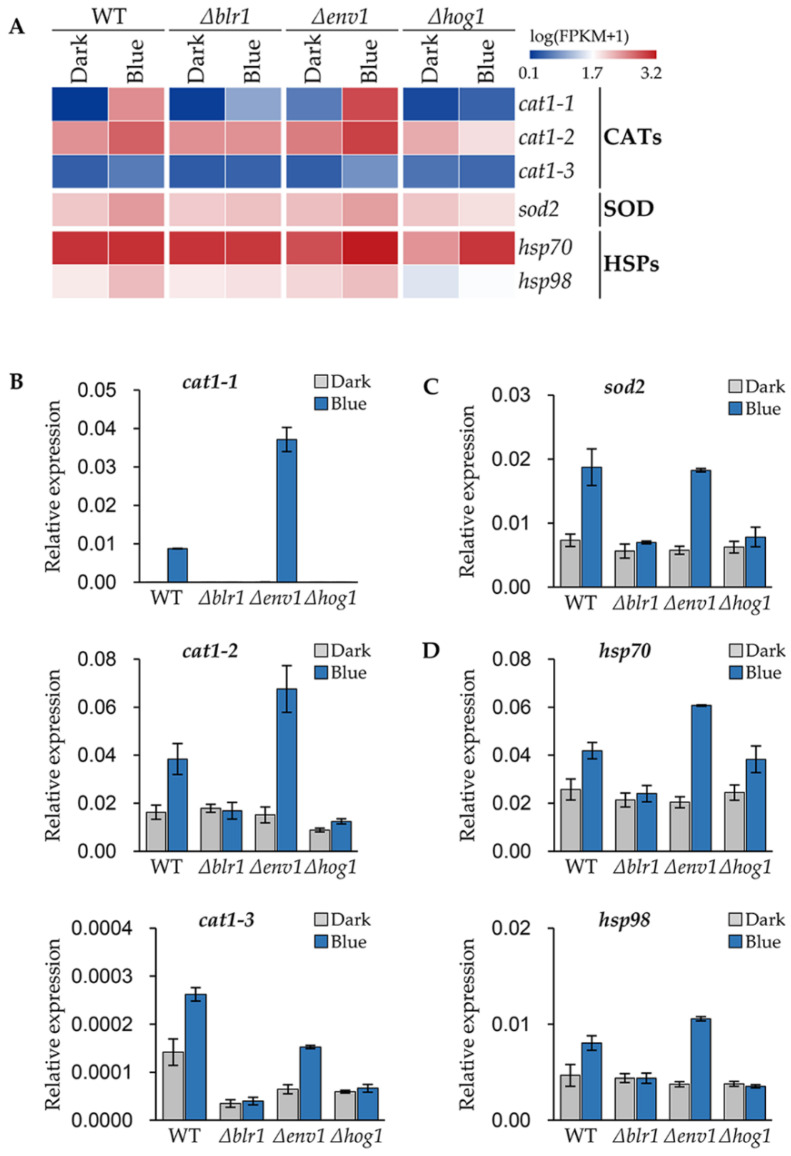
Blue-light-regulated stress response genes identified in *T. guizhouense*. (**A**) Transcriptional abundance of the catalase (CAT)-, SOD- and HSP-encoding genes. (**B**–**D**) Expression levels of the catalase- (**B**), SOD- (**C**) and HSP- (**D**) encoding genes in wild type, and the *Δblr1*-, the *Δenv1*- and the *Δhog1*-mutant strains. All strains were cultured on PDA medium at 28 °C for 24 h and then kept in the dark or exposed to blue light for 45 min. The expression level of the gene was normalized to the *tef1* gene. Error bars indicate SD of three biological replicates.

Superoxide dismutase (SOD) and catalase (CAT) are crucial for fungi to scavenge reactive oxygen species (ROS) [32]. In our previous study, 1615 blue light responsive genes in *T. guizhouense* wild type strain were identified through genome-wide gene expression analysis [24]. By reanalyzing the transcriptome data, we screened three CAT1-encoding genes, *cat1-1* (OPB39159), *cat1-2* (OPB42210) and *cat1-3* (OPB40299) and one SOD2-encoding gene, *sod2* (OPB46299) in wild type, which were upregulated significantly in blue light (Figure 6A). This result was confirmed again with qPCR in wild type, the *Δblr1*-, the *Δenv1*-, and the *Δhog1*-mutant strains. Surprisingly, in wild type the expression level of *cat1-1* in response to blue light was increased by 690 folds compared to darkness and in the *Δenv1*-mutant strain the expression level was even higher. Likewise, in blue light the expression levels of other CAT- and SOD-encoding genes were upregulated significantly in wild type and the *Δenv1*-mutant strain, but the increase was not observed in the *Δblr1*- and the *Δhog1*-mutant strains. Therefore, the upregulation of the expression of CAT- and SOD-encoding genes are controlled by blue light through BLR1 and HOG1.

Heat shock proteins (HSPs), which function in refolding misfolded proteins, can protect the cell from damages caused by a variety of environmental stresses [33]. We identified two upregulated HSP-encoding genes *hsp70* (OPB39845) and *hsp98* (OPB41524) (Figure 6A). After blue light exposure, the expression levels of *hsp70* and *hsp98* increased 63% and 72% respectively in wild type, while in the *Δenv1*-mutant strain, the expression levels of *hsp70* and *hsp98* in blue light were 3.0 and 2.8 folds of that in the dark (Figure 6D). Like the CAT- and SOD-encoding genes, the deletion of BLR1 did not cause the expression upregulation of these genes. In the *Δhog1*-mutant strain, the expression of *hsp70* only increased 57% in blue light, while *hsp98* did not change in response to blue light.

## 4. Discussion

The regulatory role of light on fungal development provided a possibility for us to enhance the conidial yield and stress resistance of *T. guizhouense*. Here, we show that blue light, superior to red, far-red and green light, can efficiently promote conidiation of *T. guizhouense*. The conidiation-related genes in *T. guizhouense*, indeed, were drastically induced by blue light [24]. The fact that conidial yield in white light was lower than in blue light also suggests some interference of different wavelengths with the blue-light response [12]. Furthermore, red and far-red light slightly promoted the conidiation, which implies that phytochrome is probably functional in *T. guizhouense*. The biological function of *Trichoderma* phytochrome is worthy of further study.

Notably, strong blue light activated the early conidiation, but inhibited the growth of vegetative hyphae of *T. guizhouense*. In *A. nidulans,* strong blue light induces the generation of Reactive Oxygen Species (ROS) in hyphae [30]. More than a moderate light signal, the high intensity blue light (≥4 μmol photons/(m^2^ × s)) is stressful for vegetative hyphae of *T. guizhouense*, which forces the shift of development from vegetative growth to asexual reproduction. However, low intensities (<4 μmol photons/(m^2^ × s)) of blue light not only increase the conidial yield of *T. guizhouense*, but these intensities do not retard the vegetative growth. During the production of conidia in the factory, in order to lower the cost and meanwhile reutilize agricultural wastes, the media for solid fermentation are commonly the discarded crop straws or other plant-derived wastes, which normally contain cellulose as main carbon source [34]. Therefore, it is important not to reduce vegetative growth to get the maximal biomass as a prerequisite for efficient cellulose degradation. Low-intensity blue light also consumes less power. Hence, low-intensity blue light stimulates conidiation, does not affect vegetative growth and is more economic. Low intensity blue light is more practical for large-scale solid fermentation.

Fungi utilize two-component system and the MAPK HOG pathway for sensing and responding to environmental signals [24,25,26,27,28,29]. The former comprises the hybrid histidine kinases (HHKs), which are potential sensors for different environmental signals, a phosphotransferase and several response regulators (RRs); the latter consists of a MAP kinase kinase kinase (MAPKKK), a MAP kinase kinase (MAPKK) and a MAP kinase (MAPK). The corresponding orthologues of the HOG pathway components in *T. guizhouense* are the MAPKKK SSK2, the MAPKK PBS2, and the MAPK HOG1. The results here reveal that in blue light, the genes encoding the components of HOG pathway were all upregulated, suggesting that the amounts of these components in conidia are probably increased, which then strengthens the HOG pathway. Hence, the conidia can also respond to other environmental signals more efficiently. The conidia formed in blue and white light were more resistant to osmotic, oxidative and pH stresses than those formed in other light conditions. Similar effects of light on conidial resistance were also observed in *M. robertsii* [23]. Our results demonstrate that light can significantly upregulate the expression of the stress-related genes, which depends on the light receptor BLR1 and the MAPK HOG pathway. Fungal hydrophobins (HFBs), the small, cysteine-rich, secreted proteins, are involved in conidial stress resistance [35]. Our recent study proved that in *T. guizhouense*, the production of hydrophobin HFB10 can be induced by blue light through BLR1 and HOG pathway. Moreover, the upregulation of cryptochrome and photolyase family proteins, severing as repair enzymes for UV–induced DNA lesions, are also under the control of BLR1 [24,36].

The blue light receptor BLR1 is essential for conidiation of *T.* *guizhouense* in blue light and controls 80% of light-regulated genes [24]. Although HOG1 controls more than 60% of light-regulated genes, the genes related to conidiation such as *brl1*, *aba1* and *wet1* are independent of it. It seems that during the process of conidiation, the role of HOG pathway is to specially modulate conidial fitness according to the intracellular and extracellular signals by equipping the conidia with stress-related proteins. Likewise, in *A. nidulans*, the viability of conidia was decreased when the MAP kinase SakA/HogA was absent [27]. HOG pathway is a hub of various environmental signals, but how it distinguishes these signals such as osmotic and oxidative stresses, light and heat shock, remains enigmatic. However, it is reasonable that a set of stress-related genes can be awoken not only by one environmental signal but others plugging into HOG pathway. That is why conidia produced in blue light are more resistant to different abiotic stresses. Recently, Rangel et al. showed that in the entomopathogenic fungi *Lecanicillium aphanocladii* and *Simplicillium lanosoniveum*, the effect of the nutritional stress on conidial resistance is stronger than that of illumination [31]. Conidia formed under nutritional stress are more resistant to several abiotic stresses than those formed in light. Whether the signal of nutritional stress is integrated into HOG pathway or there is an alternative to trigger the expression of stress-related genes remains to be elucidated in the future. The stronger effect of nutritional stress on conidial resistance also arises the question if one of the signals is overwhelming when light and other stresses coexist. Therefore, a larger regulatory network for gene expression depicting the crosstalk between light and stress signaling needs to be deciphered.

## 5. Conclusions

The strategy to improve the conidial yield and stress resistance of *T. guizhouense* through optimizing light conditions is feasible. In comparison to darkness, the conidial yield was increased by more than 1400 folds under 450 nm blue light and the conidial resistance was significantly enhanced. Low-intensity blue light (<4 μmol photons/(m^2^ × s)) improved the conidial yield and stress resistance simultaneously, but strong blue light (≥4 photons/(m^2^ × s)) delayed the vegetative growth of *T. guizhouense*. The enhanced conidial stress resistance is probably attributed to the upregulation of stress-related genes, which is controlled by the light receptor BLR1 and the MAPK HOG pathway.

## Data Availability

Not applicable.

## Supplementary Materials

The following supporting information can be downloaded at: https://www.mdpi.com/article/10.3390/jof8010050/s1. Table S1: Oligonucleotides used in this study.
